# A Cu(II)-MOF Based on a Propargyl Carbamate-Functionalized Isophthalate Ligand as Nitrite Electrochemical Sensor

**DOI:** 10.3390/s21144922

**Published:** 2021-07-20

**Authors:** Maria Cristina Cassani, Riccardo Castagnoli, Francesca Gambassi, Daniele Nanni, Ilaria Ragazzini, Norberto Masciocchi, Elisa Boanini, Barbara Ballarin

**Affiliations:** 1 Department of Industrial Chemistry “Toso Montanari”, Bologna University, Via Risorgimento 4, I-40136 Bologna, Italy; riccardo.castagnoli2@studio.unibo.it (R.C.); francesca.gambassi2@unibo.it (F.G.); daniele.nanni@unibo.it (D.N.); ilaria.ragazzini6@unibo.it (I.R.); 2 Department of Science and High Technology & To.Sca.Lab., University of Insubria, Via Valleggio 11, I-22100 Como, Italy; norberto.masciocchi@uninsubria.it; 3 Department of Chemistry “Giacomo Ciamician”, Bologna University, Via Selmi 2, I-40126 Bologna, Italy; elisa.boanini@unibo.it

**Keywords:** nitrite detection, electrochemical sensor, electroactive metal-organic frameworks, electrocatalysis

## Abstract

This paper investigates the electrochemical properties of a new Cu(II)-based metal-organic framework (MOF). Noted as Cu-YBDC, it is built upon a linker containing the propargyl carbamate functionality and immobilized on a glassy carbon electrode by drop-casting (GC/Cu-YBDC). Afterward, GC/Cu-YBDC was treated with HAuCl_4_ and the direct electro-deposition of Au nanoparticles was carried at 0.05 V for 600 s (GC/Au/Cu-YBDC). The performance of both electrodes towards nitrite oxidation was tested and it was found that GC/Au/Cu-YBDC exhibited a better electrocatalytic behavior toward the oxidation of nitrite than GC/Cu-YBDC with enhanced catalytic currents and a reduced nitrite overpotential from 1.20 to 0.90 V. Additionally GC/Au/Cu-YBDC showed a low limit of detection (5.0 μM), an ultrafast response time (<2 s), and a wide linear range of up to 8 mM in neutral pH.

## 1. Introduction

Metal-Organic Frameworks (MOFs) constituted by connecting metal ions with polytopic organic linkers have received enormous attention in these last years. Although the main research studies based on MOFs are driven by catalysis [1], gas absorption, or gas storage [2], potential uses were also proposed for electrochemical applications [1,3,4,5]. Due to the high porosity and surface area, MOF films can concentrate analytes near the surface of the electrode amplifying the signal response and improving the detection sensitivity [6,7,8,9,10]. Because MOFs suffer from low conductivity, to further increase the sensibility of the sensors in which they are employed, they are usually modified with highly conductive nanomaterials like metallic nanoparticles and carbon nanomaterials [6,11,12,13].

Nitrite is extensively present in environmental, food, industrial, and physiological systems [1] and due to their dangerousness [14,15], several analytical techniques were developed for their accurate and reliable quantification [16,17]. Among them, electrochemical sensors have received great attention for being easy to use and for presenting outstanding features such as high sensitivity, low detection limits, and good selectivity. With the aim of decreasing the high oxidation potential that nitrites show at the traditional electrodes and of lowering the detection limit, several materials with good catalytic activity have been used as electrode modifiers, e.g., graphene, carbon nanotubes, and gold nanoparticles [18,19,20,21]. A few interesting applications of functionalized MOFs for the electrochemical determination of nitrite have recently appeared [12,13,22,23], in this context, electrodes based on MOFs have been recently employed to load or encapsulate catalytically active metal nanoparticles with high catalytic efficiency [13,23,24,25].

**Figure 1:**
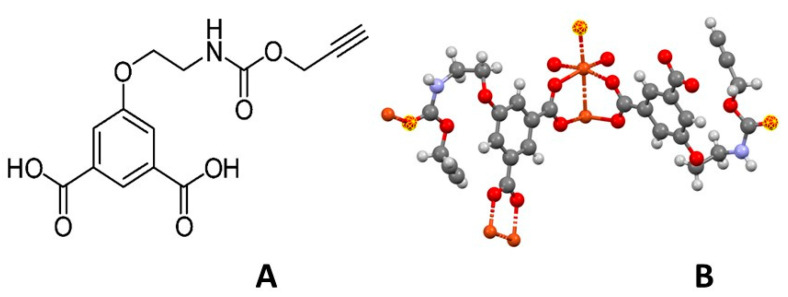
(**A**) A conventional sketch of the 1,3-H_2_YBDC moiety, showing molecular connectivity; (**B**) a 3D drawing of the Cu-YBDC paddlewheel fragment and the location of the carbamate carbonyl oxygen atoms (highlighted in yellow) completing the Cu(II) coordination through C=O^…^Cu bonds. Atomic coordinates are taken from ref. [26]. Color codes: Carbon (black); Hydrogen (white); Nitrogen (blue), Oxygen (red) and Copper (orange). The dashed bonds address the weak intermetallic interaction and the apical carbamate coordination mode.

We have recently synthesized a new Cu-MOF using the new organic linker 5-(2-{[(prop-2-yn-1-yloxy)carbonyl]amino}ethoxy)isophthalic acid shown in Figure 1A (in the 1,3-H_2_YBDC label, Y stands for alkyne and BDC for benzene carboxylate). Cu-YBDC contains a complex network of 5-substituted isophthalate anions bound to Cu(II) centers, in which the apical atom in the paddlewheel structure belongs to the carbamate carbonyl oxygen atom (Figure 1B). 

Though being this species, a full 3D MOF containing, in square channels alternating in a chessboard-like disposition [26], propargyl carbamates throughout the entire bulk, their internal accessibility is limited by the size of the pervious pores. Accordingly, only those residues, ending with a terminal alkyne function branching out from the Cu-MOF (termed Cu-YBDC) surface, may act as binding sites able to capture Au(III) ions (*vide infra*). We herein present the electrochemical characterization of Cu-YBDC and its application as a sensor. The electrode modification was obtained by drop-casting a Cu-YBDC dispersion in ethanol over a glassy carbon electrode (GC) to obtain a GC/Cu-YBDC modified electrode. Additionally, exploiting the affinity of Au(III) toward the triple bond of the propargyl group, a gold decorated GC/Cu-YBDC electrode (GC/Au/Cu-YBDC) has been prepared in two steps: first, the Cu-MOF surface was dipped in a HAuCl_4_ solution; then, the absorbed Au(III) was electrochemically reduced. The use of GC/Au/Cu-YBDC for the construction of a nitrite sensing platform was investigated.

## 2. Materials and Methods

### 2.1. Chemicals and Reagents

All reagents and solvents were purchased from commercial vendors and used as received, tris(hydroxymethyl)aminomethane hydrochloride buffer (TRIS) was purchased from Sigma Aldrich; ultrapure water purified with the Milli-Q plus system (Millipore Co, resistivity over 18 MΩ cm) was used in all cases. The detailed synthesis and characterization of Cu-YBDC are reported in our recent paper [26]. Briefly, the dicarboxylic species 1,3-H_2_YBDC was reacted with Cu(NO_3_)_2_·2.5H_2_O in refluxing 2-propanol for 24 h employing a Cu:ligand molar ratio of 1.8:1. After filtration and washing with 2-propanol, the turquoise polycrystalline powder was first dried in an oven at 70 °C for 24 h and successively kept under vacuum (0.02 bar) for 24 h. The product is stable in water at r.t and neutral pH. HAuCl_4_ was synthesized from a gold wire (BASF, 99.9999%, 1.4 mm diameter, Ludwigshafen, Germany) by dissolving the wire in hot aqua regia.

### 2.2. Apparatus and Procedure

Electrochemical techniques such as cyclic voltammetry (CV), chronoamperometry (CA), and Electrochemical Impedance Spectroscopy (EIS) were employed using a potentiostat/galvanostat Autolab GSTAT128 N (Metrohm-Autolab, Metrohm Italiana SRL, Via G. Di Vittorio, 521040 Origgio (VA), Italy) controlled by NOVA 2.10 software. The electrochemical measurements were carried out in a conventional three electrode-cell consisting of a glassy carbon (GC) working electrode (modified or unmodified; diameter 3.0 mm), a platinum wire counter electrode, and a saturated calomel reference electrode (SCE). Alternatively, a screen-printed carbon electrode (SPE) (geometric working electrode area of 3.0 mm diameter, with an Ag reference electrode and a carbon counter electrode) was used (Metrohm-C110, Metrohm Italiana SRL, Via G. Di Vittorio, 521040 Origgio (VA), Italy). The electrochemical impedance study was carried out at room temperature, at the ac voltage amplitude of 10 mV, and within the frequency range of 0.01–105 Hz in 0.1 M KCl + 2 mM K_4_[Fe(CN)_6_] solution. The equivalent circuits were obtained using ZView program analysis (available online: https://www.ameteksi.com/products/software/zview-software-en, accessed on 1 March 2020).

For XRD measurements, the GC/Cu-YBDC MOF was positioned in the diffractometer cradle using a custom-made aluminum sample holder capable of hosting small irregular specimens at the desired height. Equipment used: Diffractometer D8 ADVANCE, Bruker AXS (Bruker, Billerica, MA, USA), θ:θ configuration, Ni-filtered Cu-Kα radiation (λ = 1.5418 Å), Generator setting: 40 kV; 40 mA. Divergence slit: 0.5°; Position Sensitive Lynxeye detector. Scan-range 3–30° 2θ; Δ2θ = 0.02°, overall measuring time: ca. 16 h.

Morphological observations and energy dispersive X-ray spectrometry (EDS) analyses were carried out on uncoated specimens. A Zeiss Leo1530 Gemini (Zeiss Group, Oberkochen, Germany) field-emission scanning electron microscope was used, equipped with an InLens detector and operating at 5 kV and 15 kV, respectively. Additional information regarding the AAS and SEM characterization of the electrodes is reported in the Supporting Information.

### 2.3. Fabrication of Modified Electrodes for Electrochemical Studies

The glassy carbon electrode (GCE) was cleaned by polishing with 0.05 μm alumina slurry on a polishing cloth to create a mirror finish; the electrode was then sonicated first with absolute ethanol and then with MilliQ water (Merck Millipore, Burlington, MA, USA) for about 1.0 min, respectively. It was successively thoroughly rinsed with MilliQ water and then dried at room temperature. Analogously, the screen-printed carbon electrodes (SPE) were sonicated first with absolute ethanol and then with MilliQ water for about 1 min, respectively; after being thoroughly washed with MilliQ water they were dried at room temperature. Typically, a stable suspension of Cu-YBDC was prepared to disperse 10 mg in 8 mL of ethanol using 10 min ultrasonic agitation. Then 10 μL of this suspension (1.25 × 10^−2^ mg of Cu-YBDC, contain 1.96 × 10^−3^ mg of copper, see Supplementary Materials for details) was cast onto the electrode surface (GC/Cu-YBDC or SPE/Cu-YBDC, respectively) with a micropipette and, afterward, it was dried at 70 °C for 10 min. For the Au/Cu-YBDC electrode preparation, the GC/Cu-YBDC electrode (or SPE/Cu-YBDC) was placed in a HAuCl_4_ 5 mM ethanol solution for 10 min. The electrode was then rinsed with ethanol to eliminate the unabsorbed HAuCl_4_ and placed in a phosphate buffer (PBS 0.1 M pH 7.2 electrolytic solution). The direct electro-deposition of Au nanoparticles was carried at 0.05 V for 600 s, reducing Au(III) to Au(0). Finally, the electrode was dried at 70 °C for 10 min in the air. The amount of Au on the electrode surface is 3.75 × 10^−5^ mg (Supplementary Materials for details), corresponding to a 1.9% atomic ratio with respect to Cu sites. For comparison, the electrode termed GC/Au was obtained with the same procedure described above carried out on a bare GC electrode, i.e., without CuYBDC deposition.

## 3. Results

### 3.1. Characterization of GC/Cu-YBDC and GC/Au/Cu-YBDC Electrodes

**Figure 2:**
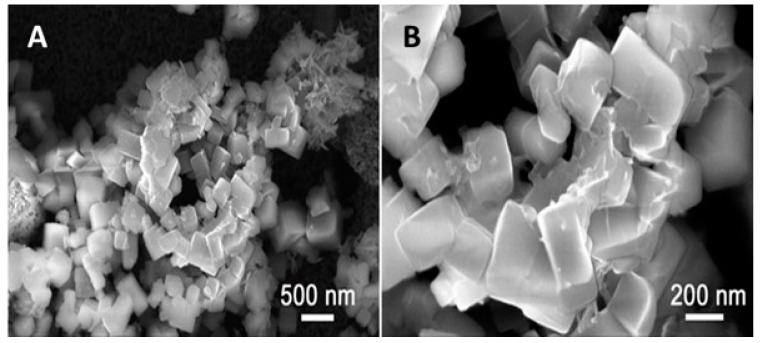
SEM images of GC/Cu-YBDC; magnified: (**A**) 50 KX and (**B**) 150 KX.

**Figure 3:**
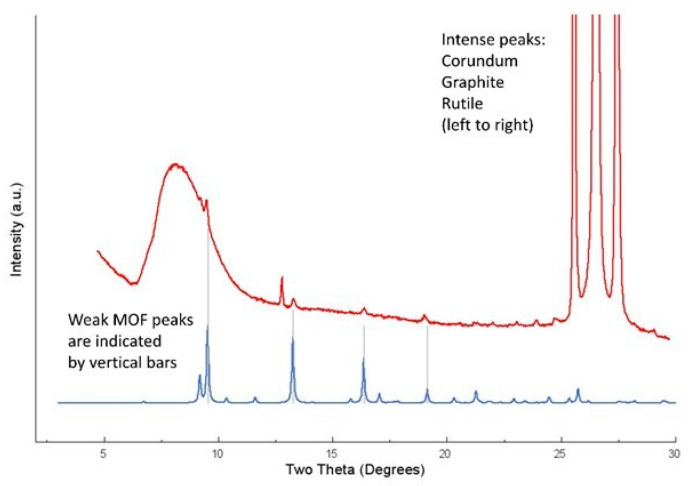
Raw diffraction data for GC/Cu-YBDC (in red) and simulated powder diffraction data from the known structure of the Cu-YBDC MOF. Vertical bars indicate the angular location of the most intense diffraction peaks of the latter. The large hump in the 7–11° 2θ range and the triplet of peaks falling above 25° 2θ stem from the sample holder (amorphous plastics and crystalline substrate, respectively).

SEM and XRD images of Cu-YBDC deposited on the electrode surface are reported in Figure 2 and Figure 3 respectively.

The obtained micrographs (Figure 2) showed no changes in the morphology of the deposited Cu-MOF with the presence of large prismatic crystals with sub-micrometric dimensions [26]; the elemental mapping (Figure S1, Supplementary Information, SI) shows the uniform presence of copper and oxygen.

A plot of raw diffraction data of GC/Cu-YBDC, compared to the powder diffraction trace simulated from the known model of Cu-YBDC, is also shown in Figure 3.

**Figure 4:**
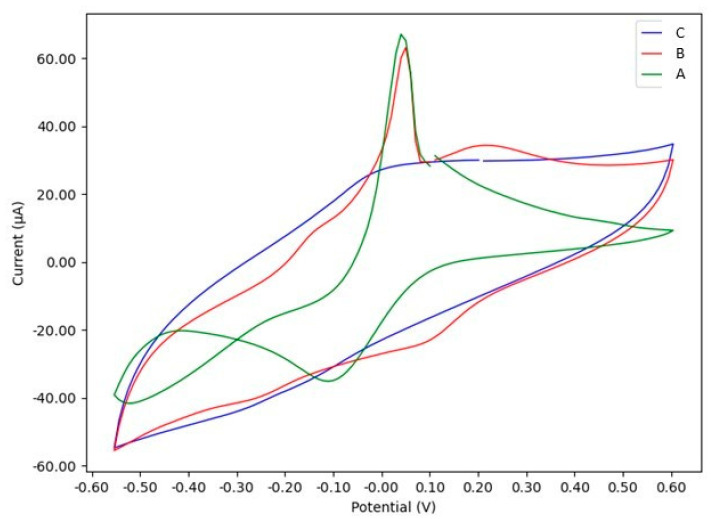
CVs of GC/Cu-YBDC at a scan rate of 50 mV/s in: A—0.1 M TRIS buffer; B—0.1 M NaCl; C—0.1 M PBS buffer electrolyte solution; the third scan is reported in all cases. The abscissa is Potential vs. SCE.

The electrochemical behavior of the GC/Cu-YBDC electrode was investigated in three commonly used electrolyte solutions: 0.1 M tris(hydroxymethyl)aminomethane hydrochloride buffer (TRIS, pH 7.2), 0.1 M NaCl, and 0.1 M phosphate buffer (PBS, pH 7.2). Figure 4, shows the *i-E* curves of GC/Cu-YBDC electrode with a potential scan in the cathodic direction starting from open circuit potential (OCP).

Usually, the electrochemical reduction of Cu^2+^ in chloride solution proceeds in two one-electron reversible waves Cu(II)/Cu(I) and Cu(I)/Cu(0), respectively, via a [CuCl_2_]^−^ intermediate. The significant stabilization of Cu(I) is favored by the presence of chlorides in the solution that leads to the formation of negatively charged Cu^I^Cl_n_ (*n* > 1) species [27]. In the case of GC/Cu-YBDC, these two reductive processes are well evident in TRIS buffer and NaCl solutions (Figure 4, paths A and B), the main difference between the CVs paths in A and B is in the peak position: 0.10 and −0.27 V for NaCl versus −0.10 and −0.52 V for TRIS electrolyte. Moreover, in the latter case a large hysteresis is observed (Figure 4, path A), highlighting that, at the inversion potential investigated, both Cu(I) and Cu(0) is still present. The more cathodic peak potential observed in TRIS may be due to the slow diffusion of the larger TRIS^+^ counter-ion during the reductive process [27]. After the potential scan is inverted, a first-week stripping anodic peak attributed to oxidation of Cu(0) to Cu(I), occurs at 0.05 V for both electrolytes, whereas the peak related to the oxidation from Cu(I) to Cu(II) is observable only with NaCl electrolyte at a potential of 0.20 V.

A completely different CV path is obtained in 0.1 M PBS solution (Figure 4, path C). In this case, in agreement with what reported in the literature, a redox couple with the oxidation and reduction peak potential at about 0.0 V, and −0.2 V, respectively, was clearly observed and was attributed to the electrochemical process occurring on Cu(II) centers [23]; the two peaks Cu(II)/Cu(I) and Cu(I)/Cu(0) overlap, as if a single redox couple was at work. No faradic signals were detected on the bare GC electrode in all the electrolyte tested solutions.

The Au/Cu-YBDC electrodes were obtained by direct reduction of the captured Au(III) into Au(0) nanoparticles in 0.1 M PBS (pH 7.2) electrolytic solution, at 0.05 V for 600 s. SEM images and the elemental maps are reported in Figure S2. It was not possible to carry out the mapping of Au, as its estimated amount is only 0.3 wt% or less (see details reported in Supplementary Materials). Nevertheless, at the highest magnification SEM image, the presence of very tiny spherical particles, were observed.

The CV paths in 0.1 M PBS buffer (pH 7.2) of GC/Au/Cu-YBDC, compared with bare GC and GC/Cu-YBDC are reported in Supplementary Materials, Figure S3. The CV curve of bare GC displays no redox peaks while the CV curve of GC/Cu-MOF shows the Cu^2+^/Cu^+^ redox reaction at a reduction peak potential of about −0.25 V and an oxidation peak potential of about −0.05 V [13]. The presence of AuNPs, with their excellent conductivity, leads to an increase of the peak current of Cu-MOF and the appearance of a new redox couple with peaks at 0.86 V and 0.41 V for oxidation and reduction potential, respectively.

**Figure 5:**
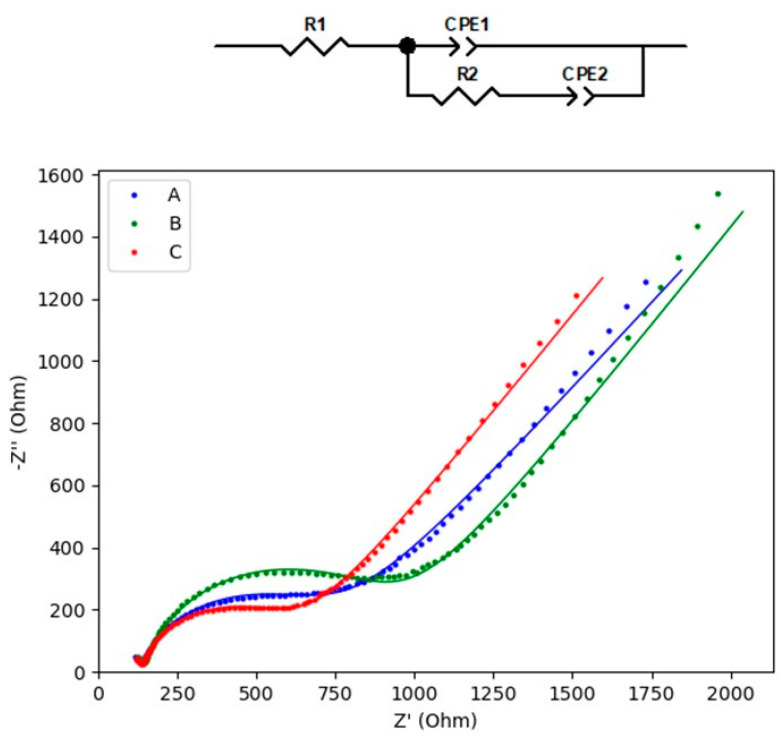
Nyquist plot obtained with A—GC, B—GC/Cu-YBDC, and C—GC/Au/Cu-YBDC electrodes recorded in 2 mM [Fe(CN)_6_]^3−/4−^ and 0.1 M KCl solution. The experimental data were well fitted (solid lines) according to the inset equivalent circuit.

Electrochemical impedance spectroscopy (EIS) was used for probing the conductivity features of the surface-modified electrodes. EIS measurements were performed in the range 10^5^ to 1 Hz at an equilibrium potential of 0.22 V. The potential amplitude applied to the electrodes was 10 mV. Figure 5 compares the Nyquist plots for bare GC, GC/Cu-YBDC and GC/Au/Cu-YBDC modified electrodes in the presence of 2 mM [Fe(CN)_6_]^3−/4−^ and 0.1 M KCl solution, recorded at the formal potential of the redox peak; the inset of Figure 5 sketches the equivalent circuit. The Nyquist plot includes the ohmic resistance, an electron transfer process located at high frequencies (kinetics), and a mass transport process in the low-frequency region (diffusion). The semicircle observed at high frequencies is due to the electron-transfer limited process and its diameter corresponds to the charge transfer resistance (R2).

The linear portion at lower frequencies is attributed to the solution resistance R1, mainly arising from the electrolyte and the intrinsic resistance of the active material. The two constant phase elements CPE1 and CPE2 (double layer capacitance at the electrode/electrolyte interface and pseudocapacitance, respectively) were employed considering the deviation from the ideal capacitor behavior (exponent n1 and n2) [27].

As expected from the electrochemical results, the R2 value follows the order: GC/Cu-YBDC > GC > GC/Au/Cu-YBDC. The higher resistance R1 and R2 values obtained for Cu-YBDC, potentially implying the worst mass transport through the material and increased resistance to electron transfer, is due to the evidence of the low porosity of the material. On the contrary, the smaller charge transfer resistance for GC/Au/Cu-YBDC is likely promoted by the synergistic effect of Cu-YBDC and the high-conductive AuNPs, which provide an enhanced conductive pathway for the electron transfer. The experimental data are well fitted according to the proposed equivalent circuit reported in Figure 5 with chi-square values of 2.3 × 10^−4^, 6.6 × 10^−4,^ and 2.3 × 10^−4^ for GC, Cu-YBDC, and Au/Cu-YBDC, respectively. The parameters obtained are reported in Table 1.

### 3.2. Electrochemical Determination of Nitrites

The overall electrochemical mechanism for nitrite ion (NO_2_^−^) involves a reversible charge transfer reaction to gives NO_2_ that subsequently disproportionate into NO_3_^−^ and NO_2_^−^. It is the last product that obeys a unidirectional reaction for the oxidation of nitrite at the Cu-MOF irreversibly modified electrodes (see Supplementary Materials, Paragraph S3 for details) [28]. To investigate the electrocatalytic performance of the different modified electrodes, the electrochemical behavior of nitrite was studied by CV technique in 0.1 M PBS buffer (pH 7.2) solution [13,23,28,29,30].

**Figure 6:**
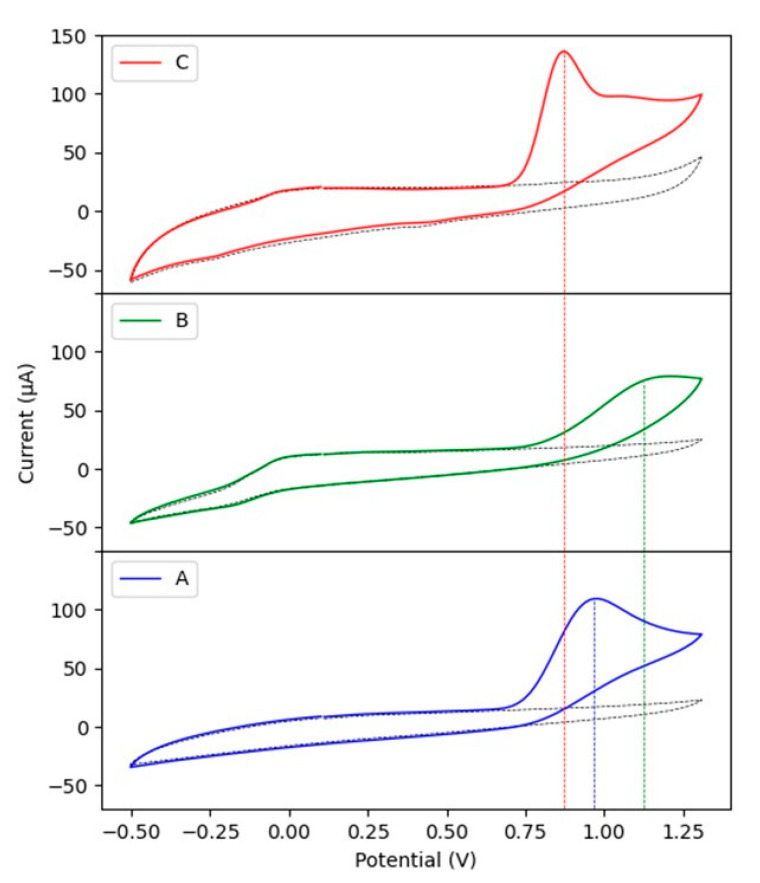
CV curves on GCE: A—bare, B—Cu-YBDC, and C—Au/Cu-YBDC in 0.1 M PBS (pH 7.2) in the absence (dashed line) and presence of 5.0 mM nitrite. Scan rate 50 mVs^−1^.

Figure 6 depicts the CV results of bare GC, GC/Cu-YBDC, and GC/Au/Cu-YBDC electrodes in 0.1 M PBS solution (pH 7.2) containing 5.0 mM nitrite. A broad oxidation hump is observed in the CV curve of bare GC (curve A) with a weak oxidation current signal and a high anodic peak potential (E_pa_ 0.99 V). A similar oxidation hump is displayed in the CV curve of GC/Cu-YBDC (curve B), but in this case, a shift towards a more oxidative potential is registered (E_pa_ 1.17 V). This behavior contrasts literature reports for Cu-MOF systems [23,24] and could be attributed to the lack of substantial porosity of Cu-YBDC that makes the modified electrode surface more resistive (as also suggested by our EIS results). Finally, the enhanced CV response at the less positive peak (E_pa_ 0.87 V), observed on Au/Cu-YBDC (curve C), is favored by the synergistic electrocatalytic effect of both Cu and AuNPs [13,23].

**Figure 7:**
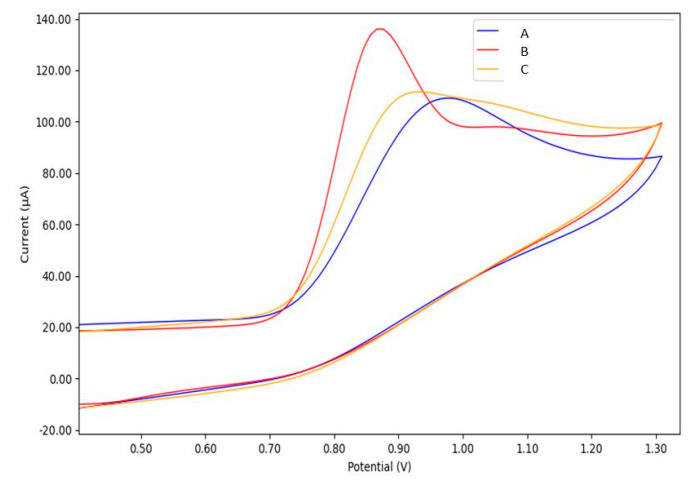
CVs of: A—bare GC, B—GC/Au/Cu-YBDC and C—GC/Au electrodes in PBS 0.1 M (pH 7.2) containing 5.0 mM nitrite; 50 mVs^−1^.

Indeed, the presence of AuNPs only on the electrode surface (GC/Au), prepared in the same way of electrode reported in Figure 6C, gives a shift towards lower oxidation peak potential, as depicted in Figure 7 (curve C).

In the composite, AuNPs accelerate the electron transfer rate in the electro-oxidation process of nitrite and lead to the decrease in the oxidation potential despite the resistive layer, in agreement with the lower resistance value (R2) observed by EIS measurements.

A study at different scan rates was conducted to determine the nature of the electrode process at the surface of the GC/Au/Cu-YBDC electrode, using CV in the 10–200 mV s^−1^ range. As shown in Figure S4, the linear dependence of the peak current intensity on the square root of scan rate demonstrates a diffusion-controlled behavior for electrooxidation of nitrite on the electrode surface. The peak potential increases with scan rate v (in a log v law), thus indicating the chemical irreversibility of the nitrite electrocatalytic oxidation process [31]. The transfer coefficient (α) can be derived from Figure S4c and the equations reported in Supplementary Materials, Figure S3 [31]. Considering the number of electrons involved in the rate-determining step as unity, a value of 0.644 was found for (1 − α), which is larger than 0.5), suggesting that the reaction intermediate is closer to nitrite than to nitrite oxidation product.

### 3.3. Chronoamperometric Determination of Nitrite on GC/Au/Cu-YBDC Sensor

**Figure 8:**
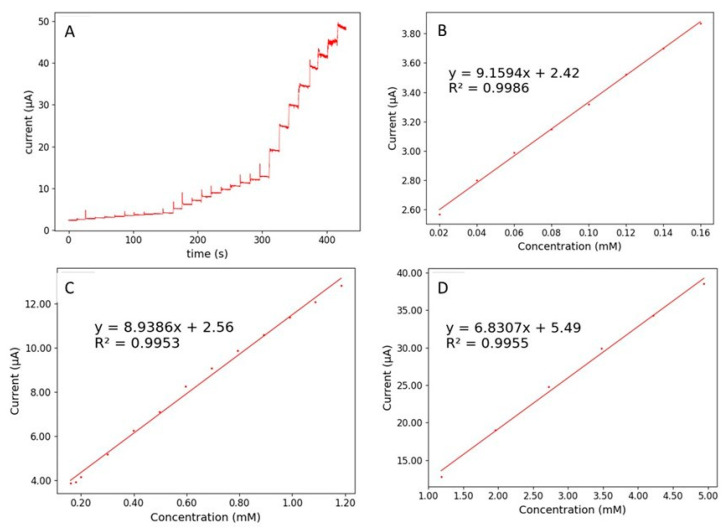
(**A**) Typical chronoamperometric response of GC/Au/Cu-YBDC to successive additions of nitrite in a mildly stirred 0.1 M PBS (pH 7.2) solution. Calibration plots of steady-state current versus nitrite concentration for three distinct ranges: (**B**) 20 μM to 160 μM; (**C**) 160 μM to 1.20 mM and (**D**) 1.20 mM to 8.0 mM.

Based on the previous characterizations, the best performances are obtained with the GC/Au/Cu-YBDC electrode. Chronoamperometry measurements were therefore performed with this electrode, with successive additions of nitrite (at a fixed potential of 0.90 V vs. SCE) in a mildly stirred 0.1 M PBS (pH 7.2) solution. Figure 8A depicts the amperometric responses of GC/Au/Cu-YBDC after progressive addition of nitrite.

Immediately after nitrite addition, a rapid increase of the current response is observed. Within 2 s or less, the current response quickly achieves 95% of the steady-state current. A linear increase of the current response with the increase of nitrite concentration was observed in different concentration regimes: from 20 μM to 160 μM (R^2^ = 0.9986); from 160 μM to 120 mM (R^2^ = 0.9953) and from 1.20 mM to 8.0 mM (0.9955), see Figure 8B–D.

The limit of detection (LOD) calculated using the formula 3σ/S, with σ being the standard deviation of the response (estimated by the standard deviation of y-intercept) and S the sensitivity values, is 5 μM, significantly lower than the maximum allowable nitrite concentration in drinking water (65 µM), as defined by the World Health Organization (WHO) [32]; accordingly, this result qualifies our sensor for such practical application, with sensitivity values of is 129.65 μA mM^−1^ cm^−2^. As expected, the chronoamperometric responses of bare GC and GC/Cu-YBDC, reported in Figure S5 and Table S1, show significantly worse sensitivity and LOD values. For the sake of comparison, the data reported in the literature for other nitrite sensors are reported in Table 2.

### 3.4. Electrochemical Stability and Reproducibility Study of GC/Au/Cu-YBDC Sensor

The reproducibility of the GC/Au/Cu-YBDC was evaluated by preparing five independent electrodes, modified under similar fabrication conditions, and testing them by cyclic voltammetry in 0.1 M PBS (pH 7.2) containing 5.0 mM nitrite. The individual current peaks for nitrite oxidation were compared and an average peak current of 1.46 × 10^−4^ A was calculated with a relative standard deviation of 4.3%. A long-term CV study was used to study the electrochemical stability of the GC/Au/Cu-YBDC sensor. No significant variation of the CV trace was observed after 15 cycles; however, a progressive decrease in the signal was observed in the subsequent fifty cycles. We attribute this observation to the formation of a high amount of copper phosphates on the surface (as imaged in Figure S6).

## 4. Conclusions

We have here studied the electrochemical behavior of the recently reported Cu(II)-MOF (Cu-YBDC), immobilized on a glassy carbon electrode by drop-casting. The modification of the GC/Cu-YBDC electrode with AuNPs was successfully obtained by the electrochemical reduction of the Au(III) ions captured by the propargyl carbamate functionality present in the linker. Such modified GC/Au/Cu-YBDC electrode has been tested as a nitrite sensor in 0.1 M PBS solution pH 7.2, exhibiting an enhanced electrochemical nitrite detection if compared to GC/Cu-YBDC. The synergic presence of copper and gold allows faster electron transport kinetics, with a calculated LOD of 5 μM, significantly lower than the maximum nitrite concentration allowed by the WHO in drinking water. Such a result qualifies this Au/Cu-YBDC sensor for being used in such an important application.

## Figures and Tables

**Table 1:** EIS parameters, (standard deviation in parentheses).

	GC	GC/Cu-YBDC	GC/Au/Cu-YBDC
R1 (Ω)	123.5 (±0.9)	137.3 (±0.8)	131.1 (±0.5)
CPE1 (Ω^−1^ s^n1^)	3.47 (±0.1) × 10^−6^	2.09 (±0.1) × 10^−6^	3.22 (±0.1) × 10^−6^
n1	0.777 (±0.004)	0.829 (±0.005)	0.817 (±0.004)
R2 (Ω)	625.0 (±7.0)	800.1 (±9.0)	502.0 (±4.5)
CPE2 (Ω^−1^ s^n2^)	2.15 (±0.002) × 10^−4^	1.84 (±0.002) × 10^−4^	2.15 (±0.002) × 10^−4^
n2	0.533 (±0.004)	0.577 (±0.006)	0.571 (±0.003)

**Table 2:** Comparison of analytic performance of different MOF material modified electrodes for nitrite detection ^a^.

Electrodes	Linearity Range (μM)	Sensitivity (μA mM^−1^cm^−2^)	LOD (μM)	Ref.
GC/Au/Cu-YBDC	20–160 160–1200 1200–8000	129.6	5.0	This work
Au/Cu-MOF/CPE	0.05–712.2	----	0.03	[23]
Au-CA/IL/Hb/CPE	5–1320	----	1.3	[33]
Cu-MOF/Au/GCE	0.1–4000 4000–10,000	----	0.092	[13]
Au/ERGO/Cu-TDPAT/GCE	0.001–1000	----	0.006	[12]
Cu-NPs/PoPD/GCE	5–22,000	----	5.0	[34]
Cu-Co/PEDOT/CNTs/GC	0.5–430	----	0.06	[35]
CuS/MWCNTs/GC	1–8000	131.2	0.33	[36]
MOX/GCE	2–120	----	0.86	[22]
MOF-525/FTO	20–800	95	2.1	[37]
Cu-MOF/rGO hybrid	3–40,000	43.7	0.033	[24]
Cu/MWCNTs/GC	5–1260	455.8	1.8	[28]

^a^ CPE = carbon paste electrode; CA = carbon aerogel: Hb = hemoglobin; IL = ionic liquid; PoPD = poly(o -phenylenediamine); CNTs = carbon nanotubes; MWCNTs = multi walls carbon nanotubes; FTO = fluorine doped tin oxide glass; rGO = reduced graphene oxide; ERGO = electrochemically reduced graphene oxide; Cu-TDPAT (H6TDPAT = 2,4,6-tris(3,5-dicarboxylphenylamino)-1,3,5-triazine).

## Data Availability

Not applicable.

## Supplementary Materials

The following are available online at https://www.mdpi.com/article/10.3390/s21144922/s1, Figure S1: SEM image (A) and elemental mapping of Cu-YBDC deposited on a SPE showing the uniform presence of: O (B) and Cu (C).Figure S2: (A) SEM images (two different magnitudes are presented) and (B) SEM and elemental mapping of Au/Cu-YBDC deposited on a SPE showing the uniform presence of O, P, and Cu. Figure S3: CV of: A—bare GC, B—GC/Cu-MOF, and C—GC/Au/Cu-MOF in PBS 0.1M (pH 7.2); 50 mV s^−1^. Figure S4: (A) CVs of GC/Au/Cu-YBDC electrode at different scan-rates from 10 to 200 mV/s in 0.1 M PBS (pH 7.2) containing 5 mM nitrite; (B) correlation between anodic peak current and square root of scan-rate; (C) correlation between anodic peak potential and logarithm of scan-rate. Figure S5: (A) Typical chronoamperometric response of bare GC (curve A) and GC/Au/Cu-YBDC (curve B) to successive addition of nitrite in a stirred 0.1M PBS (pH 7.2). (B) and (C) Calibration plots of steady state current versus nitrite concentration for the range: 0.16 to 1.20 mM and 1.20 mM to 8.0 mM. Table S1: Sensitivity and LOD values. Figure S6: SEM image of GC/Au/Cu-YBDC after 100 cycles in PBS 0.1 M pH 7.2 at a scan-rate of 50 mV s^−1^. Elemental mapping showing the uniform presence of P, O, and Cu. References [26, 31, 38, 39, 40] are cited in the Supplementary Materials.
