# Supplementary material for: A Cu(II)-MOF Based on a Propargyl Carbamate-Functionalized Isophthalate Ligand as Nitrite Electrochemical Sensor
Source: Sensors (Basel). 2021 Jul 20;21(14):4922. doi: 10.3390/s21144922 (PMC8309846; doi:10.3390/s21144922)
Supplement: Supplementary file 1 [file sensors-21-04922-s001.zip › sensors-1271394-supplementary.pdf]

sensors

Supplementary Material

A Cu(II)-MOF Based on a Propargyl Carbamate-Functionalized Isophthalate Ligand as Nitrite Electrochemical Sensor

Maria Cristina Cassani [1,*], Riccardo Castagnoli [1], Francesca Gambassi [1], Daniele Nanni [1], Ilaria Ragazzini [1], Norberto Masciocchi [2], Elisa Boanini [3] and Barbara Ballarin [1,*]

[1] Department of Industrial Chemistry "Toso Montanari", Bologna University, Via Risorgimento 4, I-40136 Bologna, Italy; riccardo.castagnoli2@studio.unibo.it (R.C.); francesca.gambassi2@unibo.it (F.G.); daniele.nanni@unibo.it (D.N.); ilaria.ragazzini6@unibo.it (I.R.)
[2] Department of Science and High Technology & To.Sca.Lab., University of Insubria, Via Valleggio 11, I-22100 Como, Italy; norberto.masciocchi@uninsubria.it
[3] Department of Chemistry "Giacomo Ciamician", Bologna University, Via Selmi 2, I-40126 Bologna, Italy; elisa.boanini@unibo.it
* Correspondence: maria.cassani@unibo.it (M.C.C.); barbara.ballarin@unibo.it (B.B.); Tel.: +39-051-2093700 (M.C.C.); Tel.: +39-051-2093704 (B.B.)

Citation: Cassani, M.C.; Castagnoli, R.; Gambassi, F.; Nanni, D.; Ragazzini, I.; Masciocchi, N.; Boanini, E.; Ballarin, B. A Cu(II)-MOF Based on a Propargyl Carbamate-Functionalized Isophthalate Ligand as Nitrite Electrochemical Sensor. *Sensors* **2021**, *21*, 4922. https://doi.org/10.3390/s21144922

Academic Editor: Alisa Rudnitskaya

Received: 7 June 2021
Accepted: 8 July 2021
Published: 20 July 2021

Publisher's Note: MDPI stays neutral with regard to jurisdictional claims in published maps and institutional affiliations.

Copyright: © 2021 by the authors. Licensee MDPI, Basel, Switzerland. This article is an open access article distributed under the terms and conditions of the Creative Commons Attribution (CC BY) license (http://creativecommons.org/licenses/by/4.0/).

1. Characterization of GC/Cu-YBDC

Assuming the homogeneity of the Cu-YBDC suspension used in drop-coating the electrodes, the amount of Cu-YBDC deposited should be 1.25×10^{-2} mg, and considering that from the elemental analysis Cu is the 15.7% of MOF [1], the amount of copper deposited on the electrodes is 1.96×10^{-3} mg. The overall amount of copper present was determined by means of flame atomic absorption spectroscopy (AAS, Thermo Scientific iCE 3300 AA01124707, Waltham, MA, USA) in an air-acetylene flame (λ= 324.8 nm; spectral bandwidth = 0.5 nm). The analyses were conducted by comparison with five calibration standards (2.0, 4.0, 6.0, 8.0, 10.0 ppm) prepared by dilution to 25 mL of different amounts of a 100 ppm standard solution prepared by diluting 1 mL of *FIXANAL* (03372-1EA Fluka, copper atomic spectroscopy standard concentrate 10.00 g/L*)* in 0.5 M HNO_3 (Normatom®, 67%–69%, d = 1.41 g/cm^3, MW 63.01, VWR Chemicals, Radnor, PA, USA,). The samples were prepared by first heating the solid (5.3 mg) with concentrated nitric acid until complete dissolution and subsequently diluted with HNO_3 0.5 M up to a volume of 100 mL.

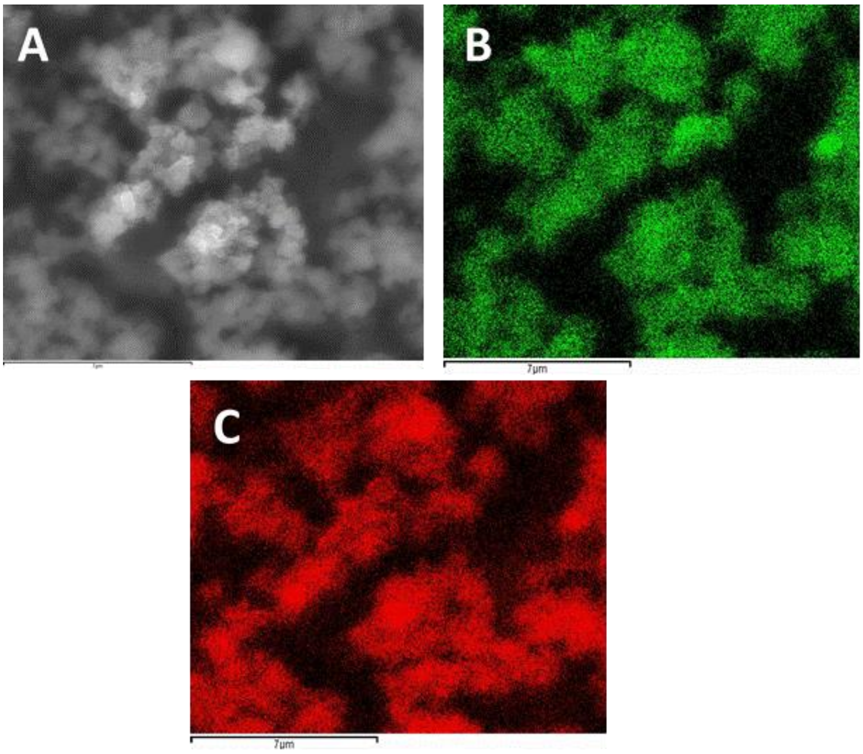

Figure S1. SEM image (**A**) and elemental mapping of Cu-YBDC deposited on a SPE showing the uniform presence of: O (**B**) and Cu (**C**).

2. Characterization of GC/Au/Cu-MOF

The amount of Au(III) adsorbed by Cu-YBDC was evaluated with the following procedure. To a suspension of Cu–YBDC (0.250 g) in ethanol, $HAuCl_4 \times 3H_2O$ (0.050 g, 0.127 mmol) dissolved in the same solvent was added. The reaction mixture was stirred for 1 h at room temperature and the solid was successively separated from the supernatant by centrifugation at 5400 rpm for 15 min. After three cycles of washings with ethanol (3 × 20 mL) and centrifugations, the product was dried at 70° C for 24 h and analyzed by means of flame atomic absorption spectroscopy (AAS, Thermo Scientific iCE 3300 AA01124707) in an air–acetylene flame (λ = 242.8 nm; spectral bandwidth = 0.5 nm). The analyses were conducted by comparison with six calibration standards (4.4, 6.6, 8.8, 11.0, 13.2, 15.4 ppm) prepared by dilution to 25 mL of different amounts of a 110 ppm standard solution prepared by dissolving 11 mg of a gold wire (BASF, 99.9999%, 1.4 mm diameter) in a minimum amount of aqua regia and then diluted with HCl 0.5 M up to a volume of 100 mL.

The samples were prepared by first heating the solid with aqua regia (HNO_3 : HCl = 1:3) until complete dissolution and subsequently diluted with HCl 0.5 M up to a volume of 100 mL. AAS analysis showed that the Cu-YBDC can seize up to ca. 0.3 wt% of gold. Considering that the amount of Cu–YBDC on the electrode surface is 1.25 × 10^{-2} mg, the amount of Au on the electrode surface is estimated to be 3.75 × 10^{-5} mg.

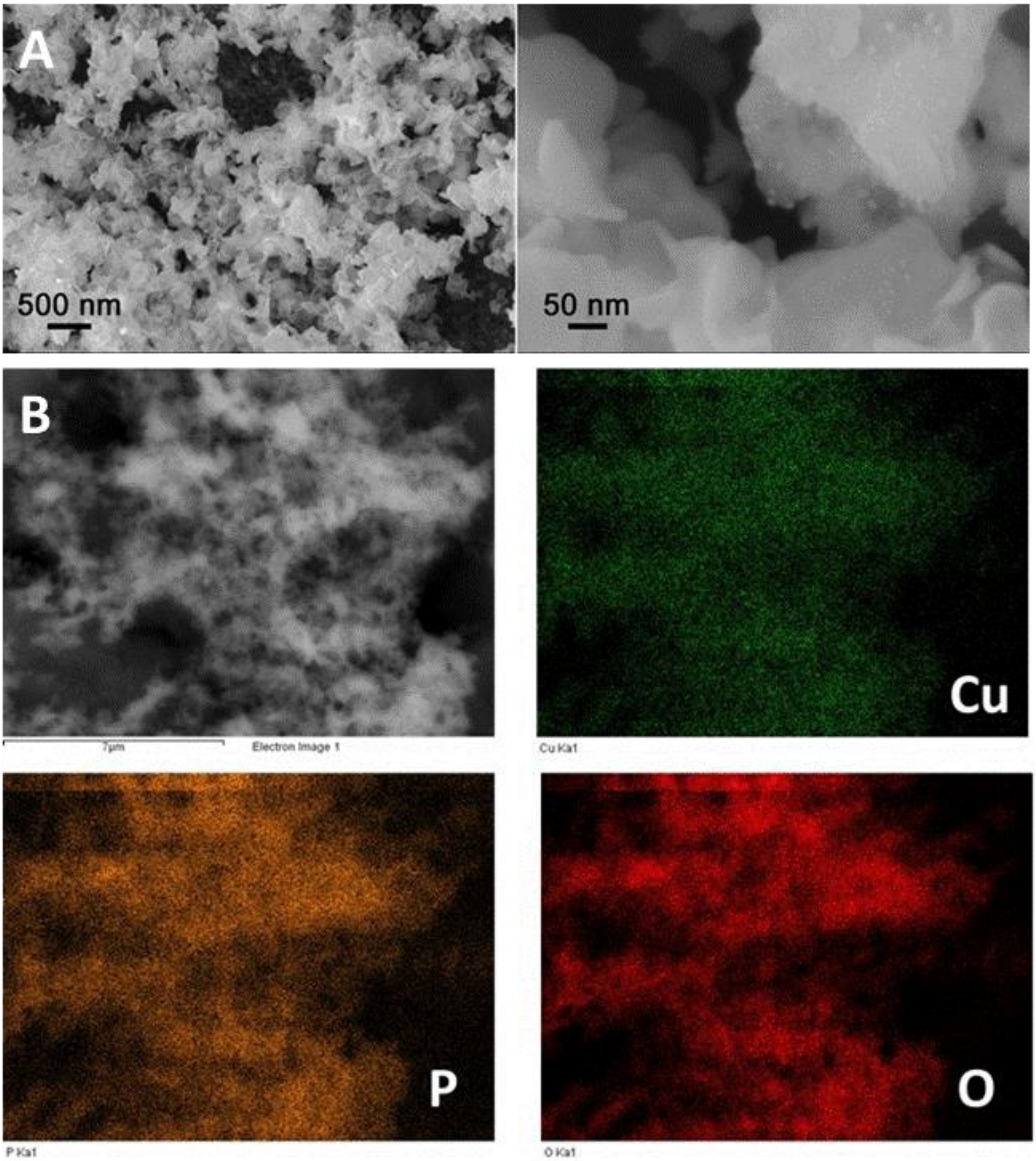

Figure S2. (**A**) SEM images (two different magnitudes are presented) and (**B**) SEM and elemental mapping of Au/Cu-YBDC deposited on a SPE showing the uniform presence of O, P, and Cu.

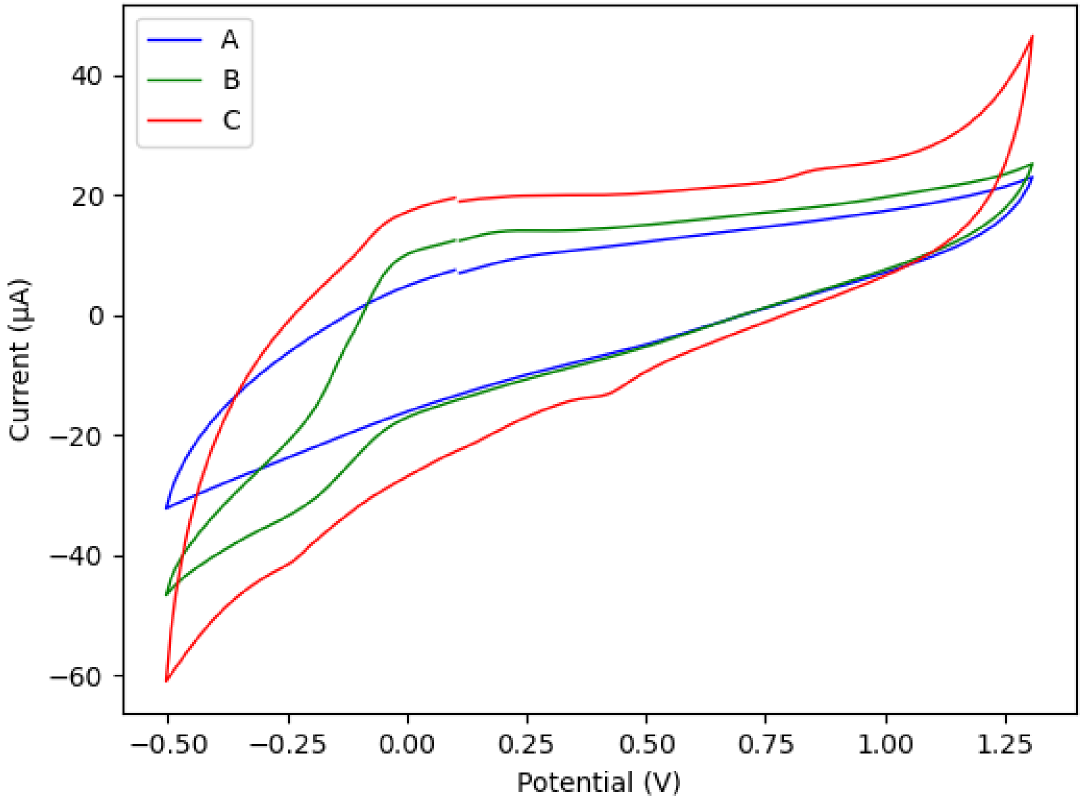

Figure S3. CV of: A – bare GC, B – GC/Cu-MOF, and C – GC/Au/Cu-MOF in PBS 0.1M (pH 7.2); 50 mV s^{-1}.

3 Nitrite Oxidation

Kozub et al. [2] reported that the nitrite oxidation is a one electron reaction that leads to nitrogen dioxide formation, which rapidly disproportionates to yield nitrite and nitrate ions in water. The EC mechanism, described in Equations (1) and (2), with a fast chemical reaction, explain the absence of a reduction peak in the CVs.

$$NO_2^- \rightleftharpoons NO_2 + e^- \quad (1)$$

$$NO_2 + H_2O \rightarrow 2H^+ + NO_2^- + NO_3^- \quad (2)$$

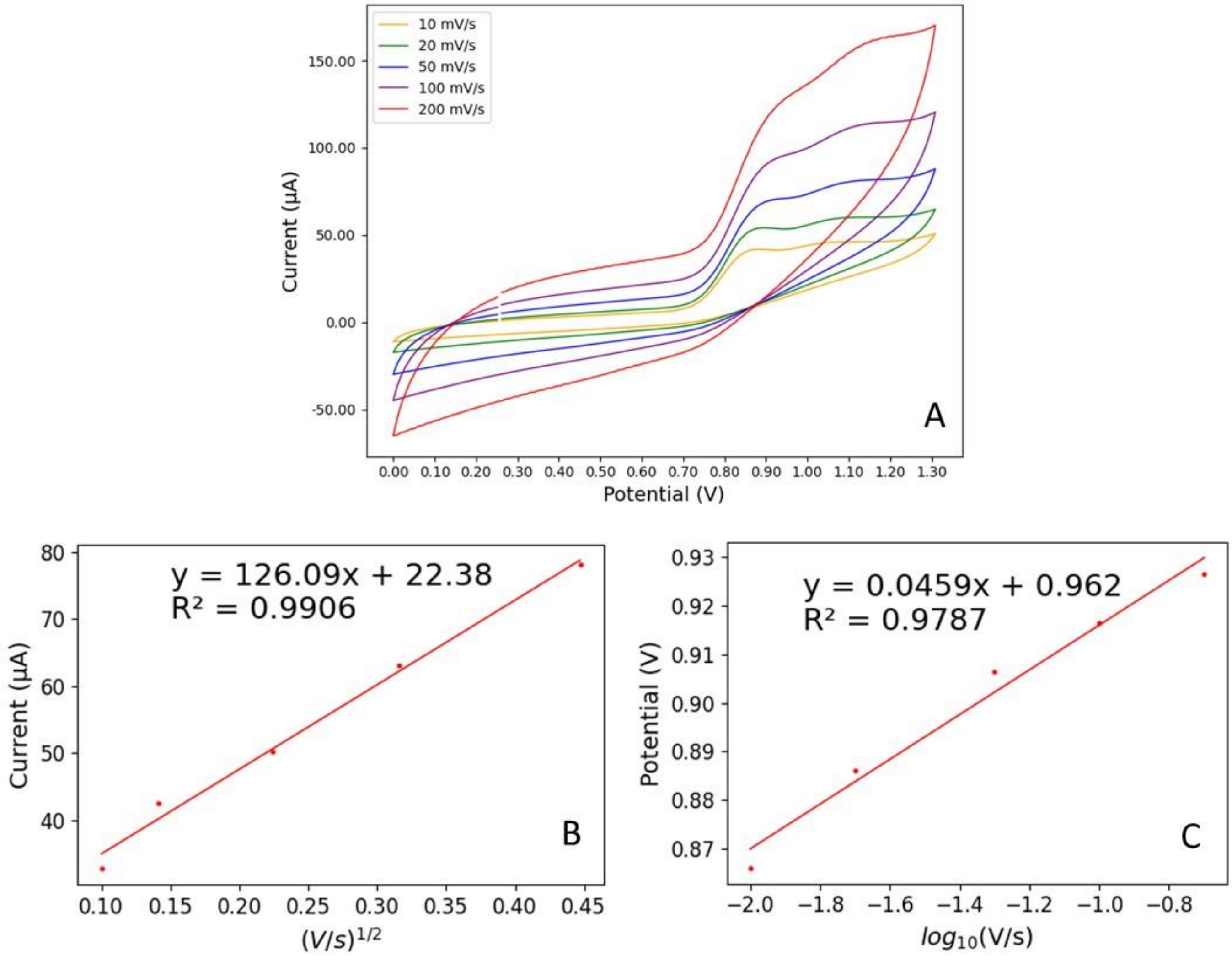

Figure S4. (**A**) CVs of GC/Au/Cu-YBDC electrode at different scan-rates from 10 to 200 mV/s in 0.1M PBS (pH 7.2) containing 5 mM nitrite; (**B**) correlation between anodic peak current and square root of scan-rate; (**C**) correlation between anodic peak potential and logarithm of scan-rate.

The Ip for the oxidation of nitrite shows a linear correlation with the $v^{1/2}$ ($R^2 = 0.9906$, slope = 0.000126 $A/\sqrt{V/s}$, intercept = 2.2×10^{-5} A) confirming a diffusive process. The Ep for nitrite oxidation slightly shifts toward higher oxidation potentials with the increase in the scan rate. Ep is linearly correlated with ln v ($R^2 = 0.9787$, slope = 0.0459 V/log(V/s), intercept = 0.962 V), meaning that the electrochemical process is controlled by the electron transfer kinetics [2]. The shift of the oxidation peak towards lower anodic potentials can be attributed to the acceleration of the charge transfer caused by AuNPs presence [3].

The transfer coefficient (α) can be derived from Figure S5C in accordance with the following Equations (Equations (1)–(5)) [4]:

$$Epa = 1 + \frac{2.303\ RT}{2(1-\alpha)nF}\log(v) \tag{3}$$

$$y = mx + q \tag{4}$$

$$y = Epa \quad m = \frac{2.303\ RT}{2(1-\alpha)nF} \quad x = \log(v) \quad q = 1 \tag{5}$$

$$0.0459 = \frac{2.303\ RT}{2(1-\alpha)nF} \tag{6}$$

$$(1-\alpha)n = \frac{2.303\ RT}{2F * 0.0459} = 0.644 \quad (7)$$

where E_{pa} is the anodic peak potential (V), I is the E_{pa}-intercept (V), R is the ideal gas constant (8.314 J/mol.K), T is room temperature (298 K), v is the scan rate (mVs^{-1}), and n is the number of electrons involved in the rate-determining step, F is the Faraday constant (96500 C mol^{-1}).

A value of 0.644 was found for $(1 - \alpha)n$ (>0.5) suggesting that the intermediate of reaction is closer to nitrite then nitrite oxidation product.

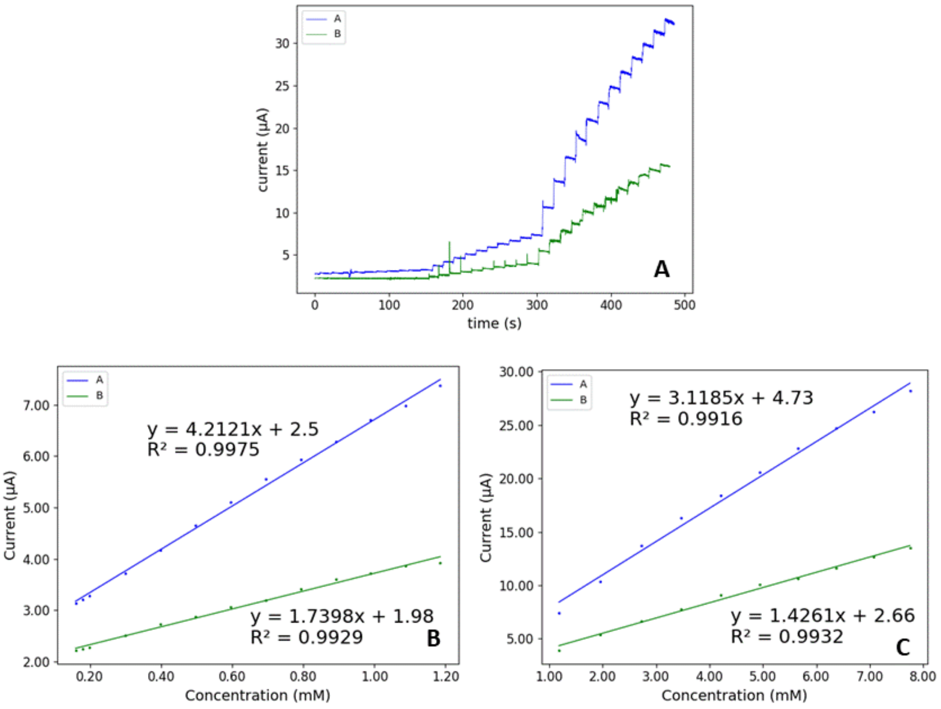

Figure S5. (**A**) Typical chronoamperometric response of bare GC (curve A) and GC/Au/Cu-YBDC (curve B) to successive addition of nitrite in a stirred 0.1M PBS (pH 7.2). (**B**,**C**) Calibration plots of steady state current versus nitrite concentration for the range: 0.16 to 1.20 mM and 1.20 mM to 8.0 mM.

Table S1. Sensitivity and LOD values.

Electrodes	Linearity Range (µM)	Sensitivity (µM mM^{-1} cm^{-2})	LOD (µM)
GC	160–12000<br>1200–8000	59.7	26
GC/Cu–YBDC	160–12000<br>1200–8000	24.6	67

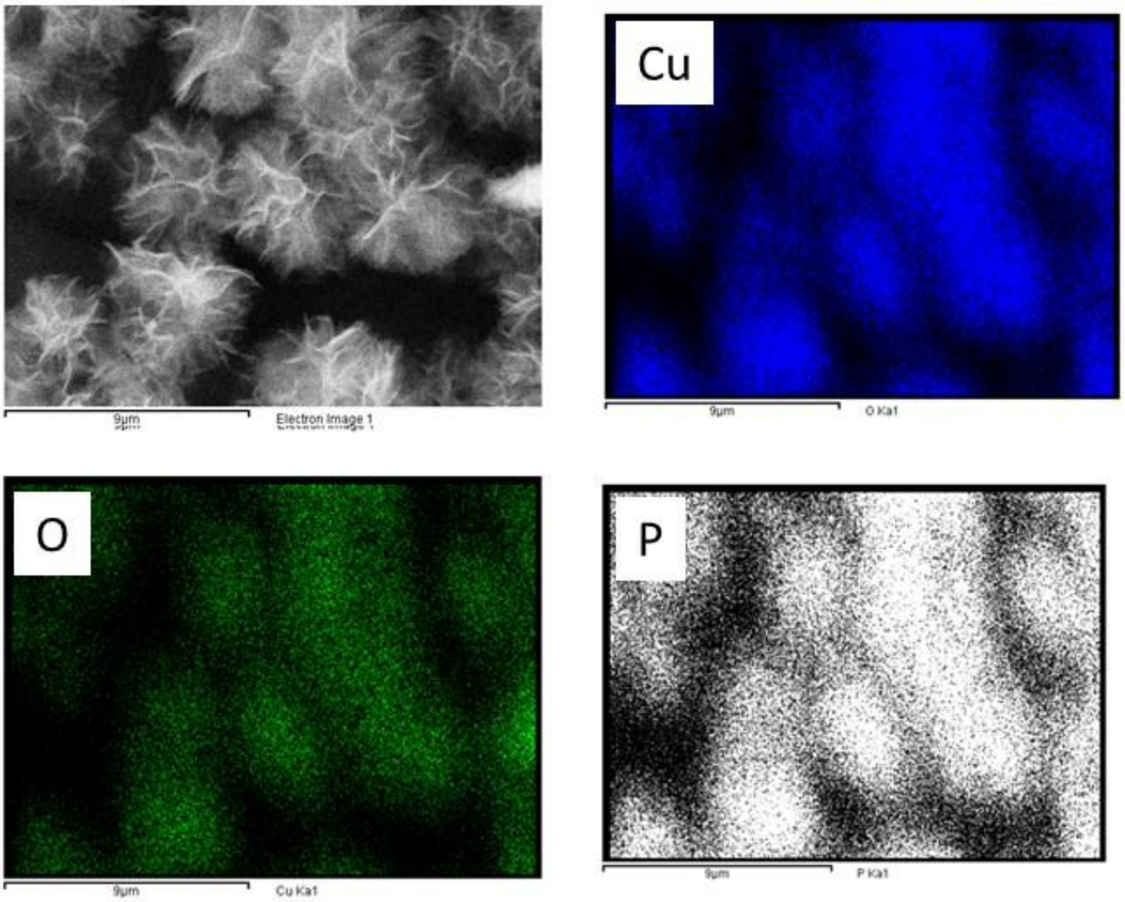

Figure S6. SEM image of GC/Au/Cu-YBDC after 100 cycles in PBS 0.1M pH 7.2 at a scan-rate of 50 mV s^{-1}. Elemental mapping showing the uniform presence of P, O, and Cu.

After being exposed to the 0.1 M PBS electrolyte solution during continuous CV cycling (100 cycles) the SEM images of the surface results very different from those of the pristine Cu-YBDC. A new nanoflower morphology appears. As reported in literature, this is typical when Cu^{2+} is added in the phosphate buffered solution as a function of the incubation time: bulk-like aggregates assembled from a large number of nanoparticles are formed immediately and with the increase in the incubation time, lamellar aggregates and then nanoflowers are observed [5]. In our case the redox cycles can accelerate this process. The EDX mapping, shown in Figure S6D, highlights the presence of phosphorus exactly in the same spots where copper and oxygen are present.

References

1. Cassani, M.C.; Gambassi, F.; Ballarin, B.; Nanni, D.; Ragazzini, I.; Barreca, D.; Maccato, C.; Guagliardi, A.; Masciocchi, N.; Kovtun, A.; et al. A Cu(II)-MOF based on a propargyl carbamate-functionalized isophthalate ligand. *RSC Adv.* **2021**, *11*, 20429–20438, doi:10.1039/d1ra02686k.
2. Kozub, B.R.; Rees, N.; Compton, R.G. Electrochemical determination of nitrite at a bare glassy carbon electrode; why chemically modify electrodes? *Sens. Actuators B Chem.* **2010**, *143*, 539–546, doi:10.1016/j.snb.2009.09.065.
3. Zhang, Y.; Li, X.; Li, D.; Wei, Q. A laccase based biosensor on AuNPs-MoS2 modified glassy carbon electrode for catechol detection. *Colloids Surfaces B Biointerfaces* **2020**, *186*, 110683, doi:10.1016/j.colsurfb.2019.110683.
4. Tau, P.; Nyokong, T. Electrocatalytic activity of arylthio tetra-substituted oxotitanium(IV) phthalocyanines towards the oxidation of nitrite. *Electrochim. Acta* **2007**, *52*, 4547–4553, doi:10.1016/j.electacta.2006.12.059.
5. Luo, Y.-K.; Song, F.; Wang, X.-L.W.; Wang, Y.-Z. Pure copper phosphate nanostructures with controlled growth: a versatile support for enzyme immobilization. *CrystEngComm* **2017**, *19*, 2996–3002, doi:10.1039/C7CE00466D.
